# Parallel evolutionary pathways to antibiotic resistance selected by biocide exposure

**DOI:** 10.1093/jac/dkv109

**Published:** 2015-05-07

**Authors:** Mark A. Webber, Rebekah N. Whitehead, Manuella Mount, Nick J. Loman, Mark J. Pallen, Laura J. V. Piddock

**Affiliations:** 1 Antimicrobials Research Group, School of Immunity and Infection and Institute for Microbiology and Infection, College of Medical and Dental Sciences, University of Birmingham, Birmingham B15 2TT, UK; 2 School of Bioscience and Institute for Microbiology & Infection, College of Life and Environmental Sciences, University of Birmingham, Birmingham B15 2TT, UK; 3 Division of Microbiology and Infection, Warwick Medical School, University of Warwick, Coventry CV4 7AL, UK

**Keywords:** disinfectant, MDR, efflux

## Abstract

**Objectives:**

Biocides are widely used to prevent infection. We aimed to determine whether exposure of *Salmonella* to various biocides could act as a driver of antibiotic resistance.

**Methods:**

*Salmonella enterica* serovar Typhimurium was exposed to four biocides with differing modes of action. Antibiotic-resistant mutants were selected during exposure to all biocides and characterized phenotypically and genotypically to identify mechanisms of resistance.

**Results:**

All biocides tested selected MDR mutants with decreased antibiotic susceptibility; these occurred randomly throughout the experiments. Mutations that resulted in de-repression of the multidrug efflux pump AcrAB-TolC were seen in MDR mutants. A novel mutation in *rpoA* was also selected and contributed to the MDR phenotype. Other mutants were highly resistant to both quinolone antibiotics and the biocide triclosan.

**Conclusions:**

This study shows that exposure of bacteria to biocides can select for antibiotic-resistant mutants and this is mediated by clinically relevant mechanisms of resistance prevalent in human pathogens.

## Introduction

Antibiotic resistance is one of the great global challenges facing modern medicine.^1,2^ Pathogenic bacteria are commonly isolated that are resistant to multiple classes of therapeutically important antibiotics and, in some cases, pan-resistant strains have arisen for which no conventional treatment remains effective.^3–5^ Antibiotic resistance can occur as a result of antibiotics exerting selective pressure leading to expansion of strains with resistance mutations or those that have acquired resistance genes via horizontal gene transfer.^6,7^ Classical experiments demonstrating the selection of antibiotic-resistant mutants have focused on antibiotic exposures to identify mutants with high-level resistance, often as a result of alterations in genes encoding the antibiotic target.^8^ However, recently it has become apparent that prolonged exposure of bacteria to antibacterial agents at concentrations below those required to arrest growth can also select for antibiotic-resistant strains. Exposure to low concentrations of drug is significant as the window of time and drug concentrations where selective pressure is exerted is much larger than in conventional experiments, where bacteria are exposed to lethal concentrations of antibiotic and only highly resistant strains can survive. When exposed to low concentrations of antibacterial, the number of potential target genes for which mutation may be beneficial and confer decreased susceptibility to antibiotics is much larger than the complement of genes that may be able to confer high-level resistance if mutated.^9,10^ Importantly, such exposure regimes allow multiple generations of bacteria to compete under low selective pressure, and this allows selection of epistatic combinations of mutations in individual strains. Individually, such mutations may have a low impact on antibiotic activity, but in combination they may have greater effects as a result of synergy. This also allows compensatory mutations to evolve in concert with resistance mutations and so minimize any fitness costs resulting from resistance.^11^

Prevention of infection is of paramount importance as the outcomes of infection with an antibiotic-resistant bacterial strain are often more severe than those of infections with antibiotic-susceptible strains.^12^ Biocides (disinfectants) are crucial to successful infection control and are widely used in hospitals, on farms, in the food industry and in the home for the control of microorganisms.^13,14^ Biocides are often mixtures of active agents that exhibit high toxicity against target organisms. As a result, it is difficult for bacteria to become resistant to the recommended in-use concentrations of many biocides as mutation within a single gene will not usually confer resistance. However, there are several examples where bacteria have become biocide resistant or show decreased susceptibility to biocides; some major mechanisms of antibiotic resistance are also relevant to bacterial survival of biocide challenge.^15^ Amongst these, MDR efflux systems can export both antibiotics and many biocides. Growth as a biofilm can also dramatically reduce the efficacy of biocide activity.^16,17^

Exposure of bacteria to biocides can select for mutants with decreased biocide susceptibility and, as we and others have seen, these mutants often display a decrease in susceptibility to various antibiotics, indicating that biocides can act as drivers of antibiotic resistance under laboratory conditions.^15,18–20^ Many common biocides are now detectable in the environment and some, such as triclosan, have been found in human urine, serum and breast milk.^21–24^ Exposure to antibiotics in the environment can result in the selection of antibiotic-resistant bacteria, which are a recognized reservoir of potential infection for humans via contamination of foodstuffs, water and surfaces with resistant pathogenic bacteria.^25,26^ The accumulation of biocides in the environment (and in vegetables and other food materials)^27,28^ also represents a potentially prolonged, low-level stress, which could exert selective pressure towards selection of bacteria with decreased biocide susceptibility, which could be cross-resistant to antibiotics. The possible results of such selective pressure are currently uncertain.

To identify the consequences of exposure of bacteria to low levels of biocide and whether there are any implications for selection of antibiotic resistance, we investigated the impact of exposure to low levels of four biocides representing different active classes on antibiotic resistance and bacterial fitness of *Salmonella enterica* serovar Typhimurium. We also used genome sequencing to determine the genetic basis for the observed resistance phenotypes.

## Methods

### Biocides and antibiotics

Four biocides representing classes with active ingredients belonging to different functional groups were chosen for use in the study: Superkill (AFS Animal Care, UK) is a mixture of aldehydes and quaternary ammonium compounds; AQAS (DuPont Animal Health) is a quaternary ammonium compound; Virkon (DuPont Animal Health) is an oxidative compound; and Trigene (Medichem International, UK) is a halogenated tertiary amine compound.

### Isolation of antibiotic-resistant mutants after exposure to biocide

*S. enterica* serovar Typhimurium strain SL1344 was the parental strain used in all experiments, representing both a pathogen and a model Gram-negative bacterium. To determine the impact of repeated, low-level exposure to biocides, SL1344 was exposed to biocides at sub-lethal concentrations that restricted growth rates to ∼70% of those in drug-free media. Therefore, Superkill and AQAS were both added to media at a concentration of 0.0018%, Trigene at 0.0005% and Virkon at 0.2%. Overnight cultures of SL1344 grown in LB broth at 37°C with shaking at 150 rpm were used to inoculate fresh, pre-warmed broth (50 μL into 5 mL) containing biocide. After 12 h of growth, a 1 mL sample was taken and stored in glycerol at −20°C and 50 μL was used to inoculate fresh media containing the same concentration of biocide (the second sub-culture). The bacterial cultures were repeatedly sub-cultured over 4 days (total of eight sub-cultures) in each biocide. Sampling was every 12 h to allow each culture to reach stationary phase before sub-culture to fresh media. A total of ∼50 generations were passaged in each experiment. In parallel to the biocide exposure experiments, SL1344 was also sub-cultured eight times in LB broth with no biocide as a control. The eight frozen samples from each biocide exposure experiment and the control experiment were serially diluted and inoculated onto antibiotic-free LB agar; after overnight incubation, these plates (with ∼200 colonies each) were replica plated onto LB agar plates, each containing a different antimicrobial at a concentration that inhibited growth of SL1344. Nalidixic acid was used at 8 mg/L, chloramphenicol at 4 mg/L, tetracycline at 4 mg/L, kanamycin at 8 mg/L and triclosan at 0.12 mg/L. After overnight incubation, colonies that grew on one or more of the antimicrobial-containing plates were selected from the original antibiotic-free LB master plate, their identity confirmed using the API 20E test (bioMérieux, France) and retained for further investigation.

### Phenotypic characterization of antibiotic-resistant mutants

The antimicrobial susceptibility of mutants resistant to one or more agents after exposure to biocide was determined for five antibiotics and five biocides using a panel of antibiotics and the standardized agar dilution method following the recommendations of the BSAC.^29^ Susceptibility to antibiotics and biocides was also determined after five passages in drug-free media to determine whether selected resistances were stable. The phenotype of the mutants in Biolog Phenomicroarray antimicrobial plates (PM11-20) was determined in duplicate and compared with that of SL1344. Data were analysed for significant differences in both replicates at multiple timepoints and in at least two sequential concentrations of test compound.

The growth rates of selected mutants were compared with that of the parental strain SL1344 by measuring the OD of cultures at 600 nm in a FLUOstar Optima reader (BMG Labtech) as previously described.^30^ The fitness costs of any of the selected mutations were evaluated in competitive index experiments by introducing SL1344 and mutants into LB broth in equal numbers and comparing the ratios of mutant to parent over time as previously described.^30^ To determine whether any mutants had changes in cellular morphology, cells of each mutant were Gram stained and visualized under a microscope.

### WGS and SNP detection

From each of the biocide exposure experiments, four drug-resistant mutants representing the range of phenotypes were selected for sequencing. Mutants were selected to reflect those that emerged early and late in the selection period. Genomic DNA was extracted from 5 mL overnight cultures grown in drug-free LB media using a Promega Wizard^®^ Genomic DNA Purification Kit. The quantity and quality of the DNA was confirmed by agarose gel electrophoresis and by analysis using a NanoDrop Spectrophotometer (Life Technologies) and Bioanalyzer (Life Technologies). Mutants were sequenced using a 454 GS FLX instrument (Roche) with Titanium chemistry and the resulting sequence reads were mapped against the SL1344 reference genome using the gsMapper alignment module of Newbler, run through the xBASE-NG web site. High-confidence SNPs were identified as previously described.^31^ Mutations detected consistently in all sequenced isolates were deemed to be differences between our WT laboratory strain and the sequenced SL1344 reference strain and were excluded from further analysis. Mutations and SNPs identified were verified by PCR and Sanger sequencing.

### Complementation of mutated genes with WT in trans and site-directed mutagenesis

The coding sequences of the WT *rpoA* and *zur* genes were amplified from SL1344 and cloned into a pBAD*myc*-HisA expression vector (Invitrogen) to create pBAD-*rpoA* and pBAD-*zur*, respectively. The plasmid pBAD-*zur* was transformed into the Trigene-selected mutants and the plasmid pBAD-*rpoA* was transformed into AQAS-selected mutants. Induction of expression of each gene was with 0.05% arabinose. RT–PCR, using primers internal to the WT gene, confirmed expression of the complements within the mutants. Site-directed mutagenesis was used to recreate the *rpoA* and *zur* changes found in the biocide exposure experiments, primers were designed to amplify the mutations found within these genes and mutagenesis was accomplished using the Red/ET recombinase kit (GeneBridges, UK) according to the manufacturer's instructions.

## Results

### Biocides select antibiotic-resistant bacteria

**Figure 1.**
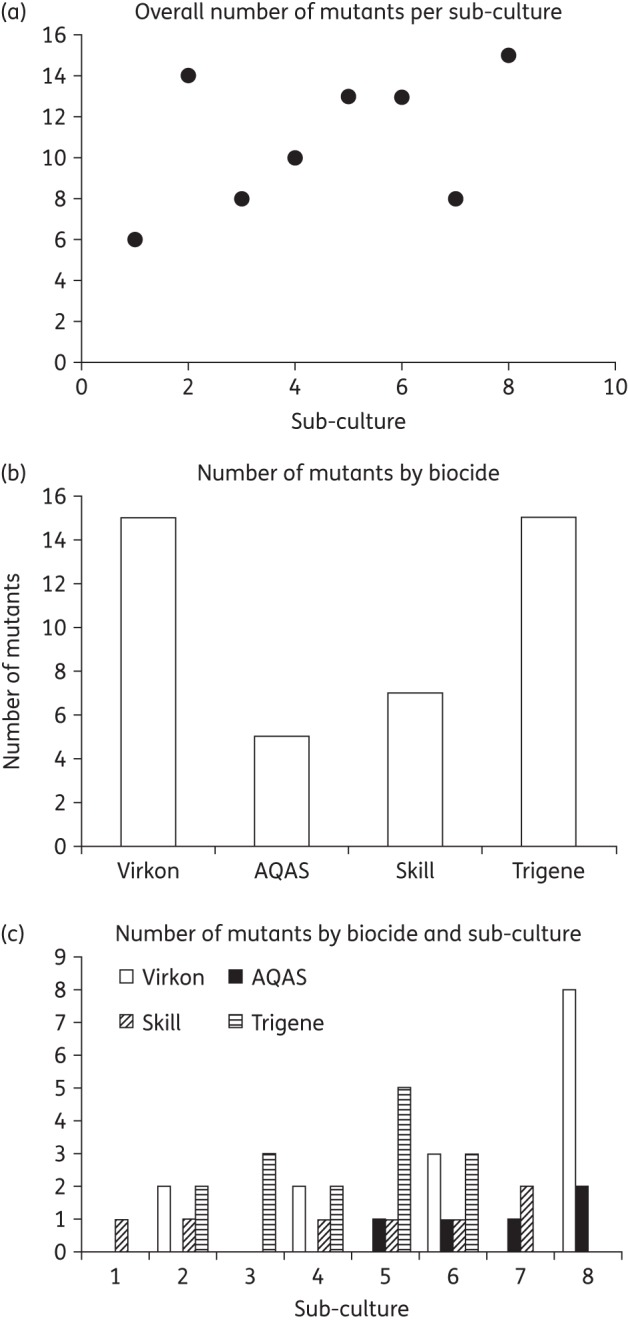
Comparison of mutant selection between sub-culture and biocide. (a) Number of mutants identified at each sub-culture. (b) Number of mutants identified after exposure to each biocide. (c) Identification of mutants per sub-culture by biocide.

Independent cultures of WT *Salmonella* Typhimurium SL1344 were repeatedly sub-cultured in subinhibitory concentrations of four biocides (Virkon, Trigene, AQAS and Superkill) with different chemistries and modes of action. Samples were taken regularly from each lineage and mutants with altered antibiotic susceptibility were identified using antibiotic indicator breakpoint plates containing concentrations of antibiotic that restrict growth of SL1344. No mutants that were retained demonstrated increased tolerance to the biocides they were exposed to, but, from as early as the second sub-culture (although this varied between biocides), all biocides selected mutants with altered susceptibility to antibiotics (Figure 1). Exposure of SL1344 to Superkill selected bacteria able to grow on plates containing nalidixic acid, chloramphenicol and tetracycline; further mutants arose sporadically throughout sub-cultures 2–8. During exposure to AQAS, bacteria able to grow on the antibiotic-containing plates were not seen until sub-culture 5. After this time, mutants were consistently recovered from the analagous set of antibiotic-containing plates as for the Superkill experiment. Sub-culture in Trigene resulted in selection of bacteria able to grow in the presence of nalidixic acid and/or triclosan from sub-culture 2 onwards; mutants with this phenotype were detected for the remainder of the experiment. In the presence of Virkon, antibiotic-resistant strains appeared on various antibiotic-containing plates after sub-culture 2; these were then detected sporadically throughout sub-cultures 2–8.

All colonies identified as having decreased susceptibility to any of the antibiotics tested were retained from the drug-free master plate and the MICs of a panel of drugs were determined (Table S1, available as Supplementary data at *JAC* Online).

The susceptibility of the majority of the Superkill and AQAS mutants (SK1, SK3, SK4, AQ2, AQ4 and AQ5) to antibiotics and biocides was similar and showed broad, decreased susceptibility to drugs of different classes (Table 1). Mutants AQ1 and SK7 had a different phenotype with decreased susceptibility to single antibiotics only. Mutants derived after exposure to Virkon demonstrated two phenotypes: resistance to quinolones and resistance to the biocide triclosan. In contrast, the mutants derived from Trigene were phenotypically identical; all mutants were highly quinolone resistant and resistant to triclosan.

**Table 1.** Antimicrobial susceptibility of representative mutants selected after biocide exposure

	Selective biocide	Sub-culture^a^	MIC (mg/L)^b^					
			NAL	CIP	CHL	TET	KAN	TRI
Strain								
SL1344	none	8	2	<0.015	1	0.5	4	0.06
AQ1	AQAS	5	**32**	0.015	2	0.5	8	0.015
AQ2	AQAS	6	**32**	0.06	**64**	**4**	4	**0.5**
AQ4	AQAS	8	**32**	0.06	**64**	**8**	4	0.25
AQ5	AQAS	8	**16**	0.03	**32**	**4**	4	**1**
SK1	Superkill	2	**32**	0.12	**64**	**8**	2	**0.5**
SK3	Superkill	5	**32**	0.12	**64**	**8**	4	**0.5**
SK4	Superkill	6	**16**	0.06	**16**	**4**	4	**1**
SK7	Superkill	7	4	<0.015	4	1	4	**4**
T2	Trigene	2	**512**	**0.25**	1	0.5	4	**>1024**
T3	Trigene	3	**512**	**0.25**	1	0.5	4	**>1024**
T6	Trigene	4	**512**	**0.25**	1	0.5	4	**>1024**
T9	Trigene	5	**512**	**0.25**	1	0.5	4	**>1024**
V2	Virkon	2	**512**	**0.25**	4	2	4	0.25
V4	Virkon	4	8	<0.015	4	2	4	**32**
V6	Virkon	6	**512**	**0.25**	4	2	4	0.25
V8	Virkon	8	**16**	<0.015	**8**	2	2	**32**
Site-directed mutants and complements								
SL1344-*rpoA*	NA	NA	**32**	0.015	2	0.5	8	0.015
SL1344-*zur*	NA	NA	2	<0.015	1	0.5	4	0.06
T2-pBAD-*zur*	NA	NA	**512**	**0.25**	1	0.5	4	**>1024**
AQ1-pBAD-*rpoA*	NA	NA	2	<0.015	1	0.5	4	0.06
AQ2-pBAD-*rpoA*	NA	NA	**32**	0.06	**64**	**4**	4	**0.5**

NAL, nalidixic acid; CIP, ciprofloxacin; CHL, chloramphenicol; TET, tetracycline; KAN, kanamycin; TRI, triclosan; NA, not applicable.

^a^Number of biocide exposures after which each strain was recovered.

^b^Values in bold indicate MICs ≥8-fold higher compared with SL1344.

Based on the phenotypes, four mutants from each biocide challenge experiment were selected for genome sequencing. The mutants were selected from a range of early and late sub-cultures to investigate whether mutations that occurred early in the selection period were maintained and whether multiple mutations accumulated during the experiments (Table 1).

### Biocide exposure selected mutations in only seven genes

In the mutants sequenced, seven genes were found to contain mutations (Table 2). Five genes were altered in more than one of the mutants, and mutations in three genes (*fabI*, *ramR* and *gyrA*) were selected after exposure to multiple biocides. The four AQAS mutants all carried an SNP in *rpoA*, encoding the RNA polymerase α subunit. Three of the AQAS mutants also had mutations in *ramR*, which encodes the local repressor of *ramA*, encoding a transcriptional activator that regulates the AcrAB-TolC MDR efflux system. Two of these mutants carried a 2 bp deletion within *ramR* and the other carried a point mutation resulting in a substitution within the DNA-binding domain of RamR. The remaining AQAS mutant, AQ1, also had a mutation in *hpaA*, a gene that codes for a putative AraC family regulatory protein. Of the four Superkill mutants selected for sequencing, three carried a 12 nt deletion in *ramR*, but for the other (SK7) insufficient depth of sequence was achieved to confidently identify mutations.

**Table 2.** Mutations identified in the genome-sequenced mutants

Strain	Gene	Start position^a^	End position^a^	Reference	Consensus	Type of mutation	Consequence	Fitness^b^
AQ1	*hpaA*	1 152 569	1 152 569	T	G	SNP	F-V	—
	*rpoA*	3 604 893	3 604 893	T	A	SNP	N294Y	
AQ2	*ramR*	638 142	638 142	C	T	SNP	G-D	—
	*rpoA*	3 604 893	3 604 893	T	A	SNP	N294Y	
AQ4	*ramR*	637 844	637 845	GT	—	deletion	loss of function	—
	*rpoA*	3 604 893	3 604 893	T	A	SNP	N294Y	
AQ5	*ramR*	637 844	637 845	GT	—	deletion	loss of function	—
	*rpoA*	3 604 893	3 604 893	T	A	SNP	N294Y	
SK1	*ramR*	637 708	637 719	GATCGCGCGCGG	—	deletion	loss of function	—
SK3	*ramR*	637 708	637 719	GATCGCGCGCGG	—	deletion	loss of function	—
SK4	*ramR*	637 708	637 719	GATCGCGCGCGG	—	deletion	loss of function	—
T2	*fabI*	1 749 266	1 749 266	G	T	SNP	G93V	0.94
	*gyrA*	2 373 804	2 373 804	T	C	SNP	D87G	
	*zur*	4 483 329	4 483 329	—	GC	insertion	loss of function	
T3	*fabI*	1 749 266	1 749 266	G	T	SNP	G93V	0.94
	*gyrA*	2 373 804	2 373 804	T	C	SNP	D87G	
	*zur*	4 483 329	4 483 329	—	GC	insertion	loss of function	
T6	*fabI*	1 749 266	1 749 266	G	T	SNP	G93V	0.94
	*gyrA*	2 373 804	2 373 804	T	C	SNP	D87G	
	*zur*	4 483 329	4 483 329	—	GC	insertion	loss of function	
T9	*fabI*	1 749 266	1 749 266	G	T	SNP	G93V	0.94
	*gyrA*	2 373 804	2 373 804	T	C	SNP	D87G	
	*zur*	4 483 329	4 483 329	—	GC	insertion	loss of function	
V2	*gyrA*	2 373 804	2 373 804	T	C	SNP	D87G	—
V4	*fabI*	1 749 266	1 749 266	G	T	SNP	G93V	—
	*avtA*	3 874 433	3 874 433	A	—	deletion	loss of function	
V6	*gyrA*	2 373 803	2 373 803	C	A	SNP	D87G	—
V8	*fabI*	1 749 266	1 749 266	G	T	SNP	G93V	—

^a^Start and end positions relevant to SL1344 genome.

^b^Calculated in competition assays; ‘—’ indicates no significant difference compared with SL1344.

All four Trigene mutants contained identical mutations in *fabI* (encoding FabI, which is involved in fatty acid biosynthesis and is the target site of triclosan). The substitution identified has previously been shown to confer triclosan resistance.^32^ The Trigene mutants also contained a 2 bp insertion within *zur*, which encodes a putative regulatory protein controlling zinc uptake systems. The Trigene mutants also contained a point mutation within *gyrA* encoding the α-subunit of DNA gyrase; the resulting substitution is known to confer quinolone resistance.^33^ The same *gyrA* mutation was also seen in both mutants selected after Virkon exposure that were quinolone resistant. The other two mutants, V4 and V8, both carried the same mutation in *fabI* as seen in the Trigene-selected mutants. Finally, one Virkon-selected mutant, V4, also carried a deletion within *avtA* encoding an alanine–valine transaminase.

The mutations identified in *ramR*, *gyrA* and *fabI* have all been extensively characterized by ourselves and others.^30,32,34^ However, there were also some novel mutations in genes not previously associated with susceptibility to antimicrobials; these included the *rpoA* mutation, seen in all the AQAS mutants, and the mutation within *zur*, seen in all the Trigene mutants.

The impact of the *rpoA* and *zur* mutations on antibiotic susceptibility in those mutants with changes in these genes were investigated both by recreating these mutations in SL1344 and by restoring the WT alleles of these genes *in trans*. A WT copy of *rpoA* was introduced into mutants AQ1, AQ2, AQ4 and AQ5 on plasmid pBAD*myc*-HisA. In the same way, a WT copy of *zur* was introduced into T2, T3, T6 and T9. Transcription of both genes was confirmed by RT–PCR and found to be similar to WT levels. When mutations were recreated in SL1344, the mutation within *zur* had no impact on antibiotic susceptibility and the *rpoA* mutation resulted in an MIC profile identical to that of AQ1 (Table 1). No differences were seen in antibiotic susceptibility between the complemented strains and the mutants, suggesting the WT *rpoA* allele is dominant to the mutant.

### Biocide-selected mutants were as fit in vitro as the parental strain

**Figure 2.**
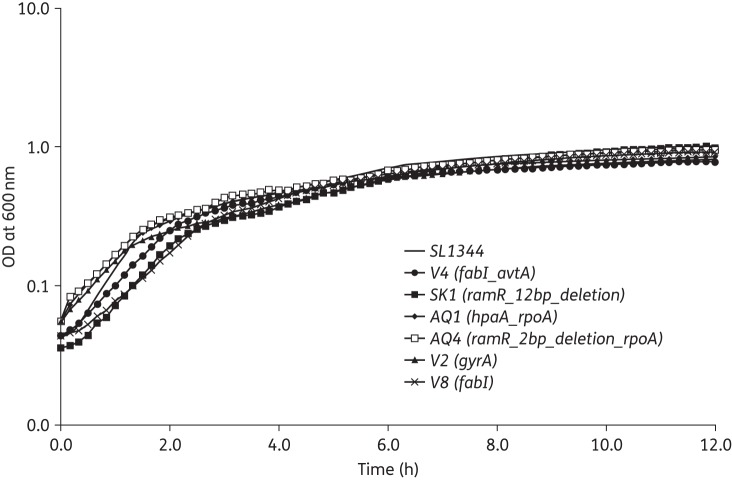
Growth kinetics of selected mutant strains in comparison with SL1344. Growth kinetics were determined by measuring the OD at 600 nm of cultures every 10 min over 16 h; data shown are the averages of eight replicate cultures for each strain.

All of the mutants were able to grow at a similar rate to SL1334; there were no statistically significant differences in generation time of final culture densities achieved (Figure 2). Mutants were also grown in competition with SL1344 after equal inoculation into drug-free media in competitive index experiments and the ratio of parent to mutant cells in the population was monitored over time. To avoid unintended impacts on fitness, mutants were differentiated from the parental strain on the basis of resistance to drugs as identified in the original screen rather than by using any additional markers. The average competitive index of the mutants relative to SL1344 ranged from 0.94 to 1.05. Only the Trigene mutants that carried a *gyrA* mutation had a competitive index value that differed significantly from SL1344 (0.94). There was no statistically significant difference between the other values for mutants compared with SL1344 (Table 2).

To further examine the possible impact of the key genotypes recovered after biocide exposure, a Biolog Phenomicroarray was used to examine the ability of each strain to respire in various concentrations of a wide panel of drugs. These experiments showed that mutation in *rpoA*, as selected after exposure to AQAS (AQ1), conferred decreased susceptibility to the quinolones enoxacin and nalidixic acid (this strain was 16-fold less susceptible to nalidixic acid compared with SL1344; Table 1) and chloramphenicol (Figure S1), but had increased susceptibility to 12 other compounds (Table S2). Inactivation of *ramR* (as seen in AQ2, AQ4, AQ5, SK1, SK3 and SK4) resulted in significantly increased respiration in the presence of seven compounds, including DNA gyrase and protein synthesis inhibitors, consistent with increased expression of MDR efflux pumps.^35^ Mutation in *fabI* was seen in mutants recovered after exposure to both Trigene and Virkon and in conjunction with mutations in *gyrA* and *zur*, or *avtA*, respectively. Mutants with either combination were better able to grow (measured by determining respiration rates) in the presence of enoxacin and, in combination with *gyrA*, this was extended to other quinolones. The *fabI* mutation in concert with *avtA* did not provide a benefit when tested with any of the other compounds in the Biolog.

Overall, these data indicate that exposure to biocides can select mutant bacteria with altered susceptibility to a wide range of antimicrobial agents.

## Discussion

Many antimicrobial products are present in the environment at low levels;^25^ here we showed that exposure to sub-lethal concentrations of all the biocides evaluated resulted in the selection of antibiotic-resistant mutants. The antibiotic susceptibility phenotypes of the mutants essentially fell into three major groups: those that were MDR; those resistant to both quinolones and triclosan; and those resistant to single agents. Surprisingly, irrespective of the biocide, exposure resulted in mutations in the same seven genes with mutations in certain genes selected for by each biocide. Both AQAS and Superkill selected for MDR mutants due to loss-of-function mutations in *ramR* that confer de-repression of the AcrAB-TolC MDR efflux pump.^35^ In addition, mutants with a substitution within GyrA at codon 87 were also selected after exposure to two of the biocides (Trigene and Virkon). This may initially seem surprising as this mutation is classically associated with resistance to quinolone antibiotics. However, we recently described a broad and generic benefit in antimicrobial survival resulting from this mutation in *Salmonella*.^30^ This also explains why it has been favoured in separate biocide exposure experiments, and this study extends the range of conditions under which this mutation is beneficial.

Both the *ramR* and *gyrA* mutations recovered after biocide exposure *in vitro* are commonly seen in clinical isolates of *Salmonella* and other Enterobacteriaceae and therefore their selection by biocide exposure is a concern. The mutations identified after exposure to diverse biocides with different mechanisms of action indicate that there are convergent pathways to the survival of antimicrobial stress present in Enterobacteriaceae, and both antibiotics and biocides can favour selection of identical mutations in the same genes (Figure S2). Mutants emerged readily after exposures to different biocides and were present after only two exposures (sub-cultures) for three of the four biocides tested. Once selected, mutants were stable; there was no evidence of accumulation of multiple mutations after further biocide exposure. The mutations selected had no great fitness costs when tested in competitive index experiments against the parental strain, and the occurrence of the *ramR*, *gyrA* and *fabI* mutations in clinical and veterinary isolates confirms that they carry no prohibitive fitness cost.^30,32,36,37^

The experimental design used maintained constant concentrations of biocides in each selection experiment (although potential degradation over each selection period was not measured), and none of the mutants identified on the basis of antibiotic tolerance showed any change in biocide MICs. These data indicate that selection of resistance to the biocides themselves did not occur under these conditions. The biocides used in this study are highly toxic to bacteria; developing resistance to these compounds would require multiple mutations and potentially a much longer selective window would be needed to allow adaptation to these compounds. However, it was evident that exposure to sub-lethal concentrations of biocide exerted selective pressure and mutations that promote bacterial survival also influenced antibiotic susceptibility. Consistent with the findings after low-level exposure in this study, we previously showed that bacteria overexpressing efflux pumps are better able to survive high concentrations of biocide.^15^ It has been demonstrated that exposure to sub-lethal concentrations of antibiotics can exert strong selective pressure and promote the rapid emergence of mutants with better ability to grow under stress than WT strains.^9,10^ Importantly, repeated exposure allows epistatic interactions to be subjected to selective pressure; this may have occurred in this study in the case of the *rpoA* and *zur* mutations. The *zur* mutation alone contributed no major discernible phenotype when complemented or recreated in isolation in the parental strain, but the *rpoA* mutation did result in decreased susceptibility to nalidixic acid and increased susceptibility to triclosan; however, this phenotype disappeared when complemented, suggesting dominance of the parental allele. In *Escherichia coli*, RpoA is known to interact with MarA and modulate its activity.^38^ In this study *rpoA* mutations analogous to those described in *E. coli* were found in concert with de-repression of *ramA*, encoding RamA, a close homologue of MarA (*ramA* is present in most Enterobacteriaceae, but not *E. coli*), so RpoA may also modulate the activity of RamA. The mutation in *zur* resulted in loss of function of Zur, a predicted transcriptional repressor. The *zur* system is involved in zinc uptake and mutations in *zur* were found in mutants with changes in both *fabI* and *gyrA*; therefore, it is not immediately clear how the resulting altered proteins may interact. In the absence of a phenotype it is possible that the *zur* mutation may be coincident and occurred in the relevant lineage before the other mutations described appeared.

Legislation concerning biocides requires proof of efficacy against target species at in-use concentrations; our data presented here indicate that sub-optimal concentrations of biocides can select common mutations that confer clinically relevant antibiotic resistance. The increasing accumulation of antimicrobial compounds, including biocides, in the environment, food and people represents a selective environment where microbes can be exposed to persistent low levels of biocides. Mutants such as those described in this manuscript could be selected in the absence of, or prior to, antibiotic treatment. Therefore, the role of environmental selection of antibiotic-resistant bacteria by biocides should be considered by government agencies as policies to control antibiotic-resistant bacteria are formulated and implemented.

## Funding

This study was supported by BBSRC grant BB/GO12016/1.

## Transparency declarations

None to declare.

## Supplementary data

Tables S1 and S2 and Figures S1 and S2 are available as Supplementary data at *JAC* Online (http://jac.oxfordjournals.org/).

Supplementary Data
