# Supplementary material for: Parallel evolutionary pathways to antibiotic resistance selected by biocide exposure
Source: J Antimicrob Chemother. 2015 May 7;70(8):2241–8. doi: 10.1093/jac/dkv109 (PMC4500774; doi:10.1093/jac/dkv109)
Supplement: Supplementary Data [file supp_70_8_2241__index.html]

Parallel evolutionary pathways to antibiotic resistance selected by biocide exposure — Parallel evolutionary pathways to antibiotic resistance selected by biocide exposure — Supplementary Data 

# Parallel evolutionary pathways to antibiotic resistance selected by biocide exposure

## Supplementary Data

Supplementary Data

- Supplementary Data - Docx file
