# Supplementary material for: Parallel evolutionary pathways to antibiotic resistance selected by biocide exposure
Source: J Antimicrob Chemother. 2015 May 7;70(8):2241–8. doi: 10.1093/jac/dkv109 (PMC4500774; doi:10.1093/jac/dkv109)
Supplement: Supplementary Data [file supp_dkv109_dkv109supp.docx]

**Supplementary data**

**Table S1. Mutants retained and characterised phenotypically**

|  |  |  | **MIC (ug/ml)** | | | | | |  | |  |
| --- | --- | --- | --- | --- | --- | --- | --- | --- | --- | --- | --- |
| **Biocide** | **Strain** | **Sub-culture** | **Chl** | **Tet** | **Tri** | **Nal** | **Kan** | **Cip** | **Stable?^a^** | **MDR?^b^** | |
| None | SL1344 | 0 | 1 | 0.5 | 0.06 | 2 | 4 | <0.015 | Yes | No | |
|  |  | 8 | 1 | 0.5 | 0.06 | 2 | 4 | <0.015 | Yes | No | |
|  |  |  |  |  |  |  |  |  |  |  | |
| Virkon | V1 | 2 | 4 | 1 | **0.5** | **32** | 4 | **0.12** | Yes | No | |
|  | V2 | 2 | 4 | 2 | 0.25 | **512** | 4 | **0.25** | Yes | No | |
|  | V3 | 2 | **8** | **4** | 0.25 | **16** | 2 | 0.03 | Yes | No | |
|  | V4 | 4 | 4 | 2 | **32** | 8 | 4 | 0.015 | Yes | No | |
|  | V5 | 4 | 4 | 1 | 0.12 | **16** | 8 | 0.06 | Yes | No | |
|  | V6 | 6 | 4 | 2 | 0.25 | **512** | 4 | **0.25** | Yes | No | |
|  | V7 | 6 | **8** | **4** | 0.25 | **16** | 2 | **0.5** | Yes | No | |
|  | V8 | 8 | **8** | 2 | **32** | **16** | 2 | 0.015 | Yes | No | |
|  | V9 | 8 | **8** | 2 | 0.25 | 8 | 4 | 0.03 | Yes | No | |
|  | V10 | 8 | **8** | 2 | 0.06 | **16** | 4 | 0.06 | Yes | No | |
|  | V11 | 8 | **8** | 2 | 0.12 | **16** | 4 | 0.06 | Yes | No | |
|  | V12 | 8 | 4 | 1 | 0.12 | 8 | 8 | 0.03 | Yes | No | |
|  | V13 | 8 | **8** | 2 | 0.12 | **16** | 2 | 0.06 | Yes | No | |
|  | V14 | 8 | 4 | 1 | 0.12 | **32** | 8 | 0.06 | Yes | No | |
|  | V15 | 8 | **8** | 2 | 0.25 | 8 | 4 | 0.03 | Yes | No | |
|  |  |  |  |  |  |  |  |  |  |  | |
|  |  |  |  |  |  |  |  |  |  |  | |
| AQAS | AQ1 | 5 | 2 | 0.5 | 0.015 | **32** | 8 | 0.015 | Yes | No | |
|  | AQ2 | 6 | **64** | **4** | **0.5** | **32** | 4 | 0.06 | Yes | Yes | |
|  | AQ3 | 6 | **32** | **8** | 0.25 | **32** | 4 | 0.06 | Yes | Yes | |
|  | AQ4 | 8 | **64** | **4** | 0.25 | **32** | 4 | 0.03 | Yes | Yes | |
|  | AQ5 | 8 | **32** | **4** | **1** | **16** | 4 | 0.03 | Yes | Yes | |
|  |  |  |  |  |  |  |  |  |  |  | |
| Superkill | SK1 | 2 | **32** | **8** | **0.5** | **32** | 2 | **0.12** | Yes | Yes | |
|  | SK2 | 4 | **16** | 2 | 0.12 | 8 | 8 | 0.06 | Yes | No | |
|  | SK3 | 5 | **64** | **8** | **0.5** | **32** | 4 | **0.12** | Yes | Yes | |
|  | SK4 | 6 | **16** | **4** | **1** | **16** | 4 | 0.06 | Yes | Yes | |
|  | SK5 | 7 | 4 | 1 | **4** | 4 | 8 | 0.03 | Yes | No | |
|  | SK6 | 7 | 2 | 0.25 | 0.03 | **32** | 8 | 0.03 | Yes | No | |
|  | SK7 | 7 | **16** | 1 | **4** | 4 | 4 | 0.015 | Yes | No | |
|  |  |  |  |  |  |  |  |  |  |  | |
| Trigene | T2 | 2 | 1 | 0.5 | **>1024** | **512** | 4 | **0.25** | Yes | No | |
|  | T3 | 3 | 1 | 0.5 | **>1024** | **512** | 4 | **0.25** | Yes | No | |
|  | T6 | 4 | 1 | 0.5 | **>1024** | **512** | 4 | **0.25** | Yes | No | |
|  | T9 | 5 | 1 | 0.5 | **>1024** | **512** | 4 | **0.25** | Yes | No | |
|  | T14 | 6 | **8** | 1 | **>1024** | **512** | 1 | **0.25** | Yes | No | |

Chl = chloramphenicol; Tet = tetracycline; Tri = triclosan; Nal = nalidixic acid; Kan = kanamycin; Cip = ciprofloxacin.

Values in bold are 8 X or more higher than the corresponding SL1344 value.

^a^ stable MIC after two drug free passages, b MDR defined as MICs of at least three drugs of separate class 8 X higher than SL1344 MIC

**Table S2. Compounds in which mutants showed a significantly different respiratory activity to SL1344 in Biolog experiments**

|  |  | Strain/genotype | | | | |
| --- | --- | --- | --- | --- | --- | --- |
| Compound | Mode of action | AQ1 (*rpoA*) | | T6 (*gyrA/fabI*) | SK3 (*ramR*) | V4 (*fabI/avtA)* |
| Furaltadone | DNA damage | ↓ | - | | - | ↓ |
| Nitrofurantoin | DNA damage | ↓ | - | | - | - |
| Acriflavine | DNA intercalation | ↓ | - | | - | - |
| Enoxacin | Topoisomerase inhibitor | ↑ | ↑ | | ↑ | ↑ |
| Nalidixic acid | Topoisomerase inhibitor | ↑ | | ↑ | ↑ | ↓ |
| Novobiocin | Topoisomerase inhibitor | - | | - | ↑ | - |
| Iodoacetate | Oxidation | ↓ | | - | - | - |
| Chelerythrine | Protein kinase C inhibitor | ↓ | | - | - | - |
| Chloramphenicol | Protein synthesis inhibitor | ↑ | | - | ↑ | - |
| Sisomicin | Protein synthesis inhibitor | ↓ | | - | - | - |
| Demeclocyline | Protein synthesis inhibitor | ↓ | | - | ↑ | - |
| Doxycycline | Protein synthesis inhibitor | ↓ | | - | ↑ | - |
| Rifampicin | RNA polymerase inhibitor | ↓ | | - | - | - |
| Potassium tellurite | Toxic anion | ↓ | | - | - | - |
| Thallium acetate | Toxic cation | ↓ | | - | - | - |
| Cupric chloride | Toxic cation | - | | - | - | - |
| Amoxicillin | Cell wall synthesis inhibitor | - | | - | ↑ | - |
| Cloxacillin | Cell wall synthesis inhibitor | ↓ | | - | - | - |

| ‘↑’ and ‘↓’indicate mutants with significantly better or worse respiration than SL1344 in the corresponding drug, respectively. No significant change is marked by an ’–‘. |
| --- |

**Figure S1. Differential metabolic activity of mutant with *rpoA* change.**

**
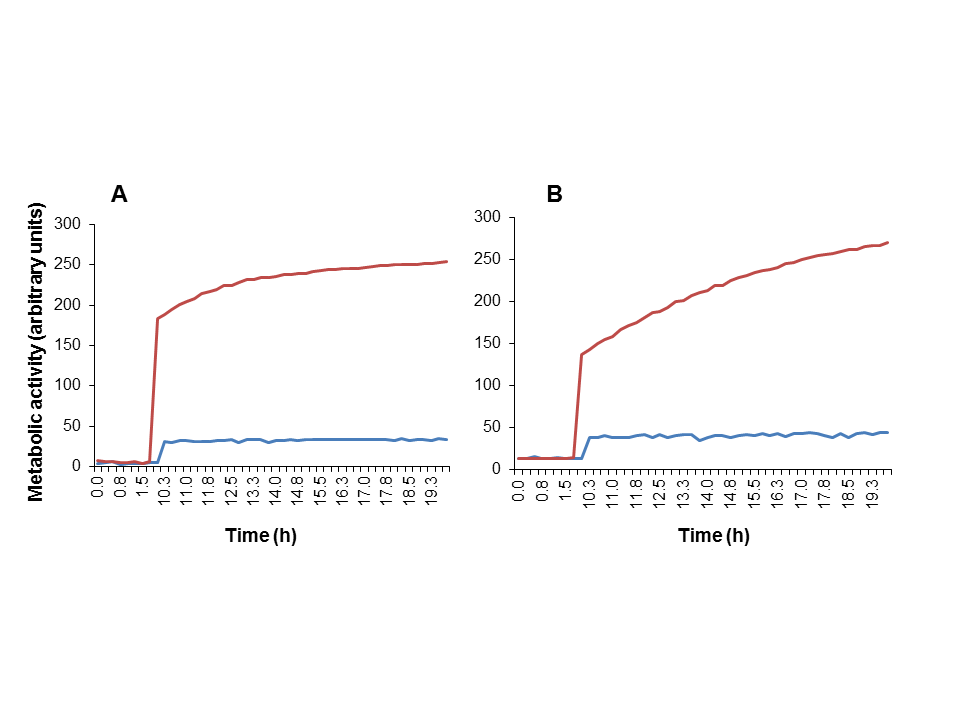
**

**Panel A shows metabolic activity over time in the presence of enoxacin, panel B chloramphenicol (both present within the media at 16 μg /ml). Data from SL1344 represented by blue lines, data from AQ1 (with mutation within *rpoA*) represented by red lines.**

**Figure S2.** **Summary of mutations present in antibiotic resistant mutants and affected pathways relevant to antibiotic resistance.**

**
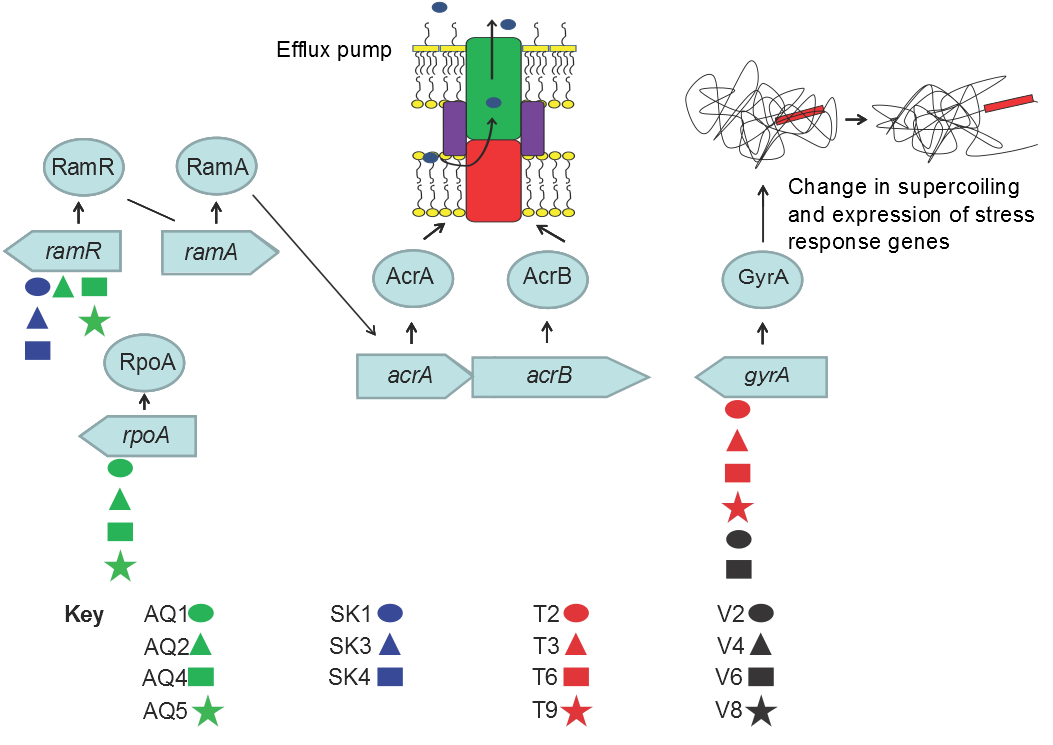
**

An overview of the sites where mutations were found, and their known roles in antibiotic resistance illustrating the overlap between mutants selected by different biocides and the convergent nature of the genes in which mutations were selected.
